# How to select outcome measurement instruments for outcomes included in a “Core Outcome Set” – a practical guideline

**DOI:** 10.1186/s13063-016-1555-2

**Published:** 2016-09-13

**Authors:** Cecilia A. C. Prinsen, Sunita Vohra, Michael R. Rose, Maarten Boers, Peter Tugwell, Mike Clarke, Paula R. Williamson, Caroline B. Terwee

**Affiliations:** 1 Department of Epidemiology and Biostatistics, EMGO Institute for Health and Care Research, VU University Medical Center, De Boelelaan 1089a, 1081 HV, Amsterdam, The Netherlands; 2 Department of Pediatrics, Faculty of Medicine and Dentistry, University of Alberta, Edmonton, AB Canada; 3 School of Public Health, University of Alberta, Edmonton, AB Canada; 4 Women’s and Children’s Health Research Institute, University of Alberta, Edmonton, AB Canada; 5 Department of Neurology, King’s College Hospital, London, UK; 6 Amsterdam Rheumatology & Immunology Center, Amsterdam, The Netherlands; 7 Department of Medicine, University of Ottawa, Ottawa, ON Canada; 8 Northern Ireland Network for Trials Methodology Research, Institute of Clinical Sciences, Royal Hospitals, Queen’s University Belfast, Belfast, UK; 9 Department of Biostatistics, University of Liverpool, Liverpool, UK

**Keywords:** COMET, Core Outcome Set, COSMIN, Delphi study, Guideline, Instrument selection, Outcomes research, Outcome measurement instrument

## Abstract

**Background:**

In cooperation with the Core Outcome Measures in Effectiveness Trials (COMET) initiative, the COnsensus-based Standards for the selection of health Measurement INstruments (COSMIN) initiative aimed to develop a guideline on how to select outcome measurement instruments for outcomes (i.e., constructs or domains) included in a “Core Outcome Set” (COS). A COS is an agreed minimum set of outcomes that should be measured and reported in all clinical trials of a specific disease or trial population.

**Methods:**

Informed by a literature review to identify potentially relevant tasks on outcome measurement instrument selection, a Delphi study was performed among a panel of international experts, representing diverse stakeholders. In three consecutive rounds, panelists were asked to rate the importance of different tasks in the selection of outcome measurement instruments, to justify their choices, and to add other relevant tasks. Consensus was defined as being achieved when 70 % or more of the panelists agreed and when fewer than 15 % of the panelists disagreed.

**Results:**

Of the 481 invited experts, 120 agreed to participate of whom 95 (79 %) completed the first Delphi questionnaire. We reached consensus on four main steps in the selection of outcome measurement instruments for COS: Step 1, conceptual considerations; Step 2, finding existing outcome measurement instruments, by means of a systematic review and/or a literature search; Step 3, quality assessment of outcome measurement instruments, by means of the evaluation of the measurement properties and feasibility aspects of outcome measurement instruments; and Step 4, generic recommendations on the selection of outcome measurement instruments for outcomes included in a COS (consensus ranged from 70 to 99 %).

**Conclusions:**

This study resulted in a consensus-based guideline on the methods for selecting outcome measurement instruments for outcomes included in a COS. This guideline can be used by COS developers in defining *how* to measure core outcomes.

**Electronic supplementary material:**

The online version of this article (doi:10.1186/s13063-016-1555-2) contains supplementary material, which is available to authorized users.

## Background

There is a lack of consensus with regard to the selection of outcomes (i.e., constructs or domains) and outcome measurement instruments (OMIs) for clinical trials [1]. As a result, different outcomes are assessed and a variety of OMIs (e.g., assessments by health professionals, biomarkers, clinical rating scales, imaging tests, laboratory tests, patient questionnaires, and performance-based tests) measure the same outcome, causing inconsistencies in reporting and difficulties in comparing and combining the findings in systematic reviews and meta-analyses [2, 3]. In addition, the quality of OMIs varies considerably, and it is usually not apparent that the most reliable and valid OMI has been selected. Standardization of the selection of outcomes and OMIs is needed.

The current project is a joint initiative between the COnsensus-based Standards for the selection of health Measurement INstruments (COSMIN) initiative [4] and the Core Outcome Measures in Effectiveness Trials (COMET) initiative [5]. COSMIN aims to improve the selection of OMIs, and has developed methodological standards for studies on the measurement properties of OMIs [6]. COMET aims to facilitate the development and application of agreed standardized sets of outcomes, also known as “Core Outcome Sets” (COS). A COS is an agreed minimum set of outcomes that should be measured and reported in all clinical trials of a specific disease or trial population. It is a recommendation of *what* should be measured and reported in all clinical trials [7].

Once a COS is defined, it is then important to achieve consensus on *how* these outcomes should be measured, i.e., which OMIs should be selected. In the selection of OMIs, a number of tasks need to be performed. For example, a literature search to find potentially relevant OMIs, and a quality assessment to evaluate the (methodological) quality of the available OMIs. However, no guidelines are currently available to support OMI selection in a standardized and rigorous way [8].

The primary aim of this study was to develop a guideline on how to select OMIs for outcomes included in a COS. However, a COS is not usually specific for any given clinical trial. A clinical trial may impose additional requirements for selecting OMIs perhaps relating to feasibility or sensitivity. We therefore had a secondary aim of investigating whether the methods for selecting OMIs for a COS are similar to the methods for selecting OMIs for individual clinical trials.

## Methods

As details on the methods and design have been published previously [9], this section is restricted to a summary.

### Study design

A Delphi study was performed to achieve consensus on relevant tasks that need to be performed in the process of selecting OMIs for outcomes (i.e., constructs or domains) included in a COS. The resulting guideline is based on the results of the Delphi study. Also, existing methodology that has been developed by COSMIN for performing systematic reviews of OMIs was used to support the guideline [4] as well as methodology that stems from the Outcome Measures in Rheumatology (OMERACT) Filter 2.0 and the *OMERACT Handbook* for developing COSs for rheumatic diseases [10, 11], and the Primary Outcomes Reporting in Trials (PORTal) initiative which looks at primary outcomes reported in adult and pediatric clinical trials [12]. These other sources of evidence were used to expand on items to a level not discussed in the Delphi study.

### Literature review

To inform the Delphi study, a literature review was performed to identify existing studies that provide guidance on OMI selection. A health research librarian conducted an electronic literature search in November 2012 in MEDLINE, EMBASE, PsycINFO, and Cinahl.

Inclusion criteria: studies that were guidelines, meta-analyses, review articles, or systematic reviews, and study protocols that developed or applied methodology for selecting outcomes or OMIs to be used in clinical trials. Exclusion criteria: studies that discussed “how to measure” rather than “how to select” outcomes or OMIs for use in clinical trials; and studies that aimed to evaluate the measurement properties of OMIs.

All search strategies are presented in Additional file 1.

### Development of the Delphi questionnaire

The potentially relevant tasks on OMI selection identified from the literature review were included in the Delphi questionnaire. Questions were formulated on the relevance of each of the tasks, for example: “Should COS developers agree upon the target population before starting to search for outcome measurement instruments?” Response options included “highly recommended,” “desirable,” “not relevant,” and “not my expertise.” Free text boxes were included after each question to facilitate comments.

### Selection of experts

Experts who were identified from the literature review, as well as experts who participated in a previous COSMIN Delphi study [13], were invited to participate. A “snowball sampling” approach was used to identify other potential experts. We found no guidelines for sample sizes of Delphi studies, but in general having more panelists will facilitate acceptance and implementation of the guideline [14]. Based on our previous experiences with Delphi studies [6, 13, 15, 16], we anticipated a response rate of between 30 and 40 %. We therefore invited all 481 previously identified experts to participate.

### Delphi rounds

The Delphi study was planned to consist of three questionnaire rounds in order to achieve consensus [17]. Panelists were asked to anonymously rate the relevance of different tasks on OMI selection. They were encouraged to justify their choices and to add other possibly relevant tasks. Subsequently, panelists were asked for their opinion on whether the methods for selecting OMIs for a COS are similar to the methods for selecting OMIs for individual clinical trials.

Consensus was defined as being achieved when at least 70 % of the panelists agreed with a task (i.e., highly recommended or desirable) with no opposing arguments provided, and when fewer than 15 % of the panelists disagreed with a task (i.e., “not relevant”). Tasks on which such consensus was reached were included in the guideline and panelists were not asked to vote for these tasks again. When at least 50 % of the panelists disagreed with a task (i.e., “not relevant”) and when no strong arguments in favor of this task were given, we excluded the task from the guideline. Tasks with an indeterminate response were taken to the subsequent round. When consensus was not reached after the third round, the need for a fourth questionnaire round was considered by the Delphi Steering Committee (CP, SV, MR, and CT).

### Data analysis

Data were analyzed both quantitatively (absolute values, percentages) and qualitatively (listings of the comments and suggestions given by the panelists). Based on the responses given in the first round, including the comments given in the free text boxes, new proposals were formulated. Response options included –“strongly agree,” “agree,” “no opinion,” “disagree,” and “strongly disagree.” Additionally, new questions that arose based on the comments given were formulated and were marked as “new questions.” Panelists were asked to rate their agreement on the given proposals and the relevance of the new tasks in the second round. The results of the second round were then again analyzed for consensus following the same procedure as for the first round [9].

## Results

### Study population

A total of 481 experts were invited to participate. Delivery failed to 41 recipients and four “out of office” notifications were received concerning long-term absence. A total of 120/436 panelists (28 %) accepted the invitation. 95/120 panelists (79 %), from 14 different countries, completed the first Delphi questionnaire (Table 1). The second questionnaire was completed by 65/95 (68 %), and 76/95 (80 %) completed the third questionnaire.

**Table 1:** Characteristics of the panelists

Study characteristics	Panelists (*N* = 95)^a^
Country, number (%)	
Australia	15 (16)
Canada	14 (15)
Denmark	7 (7)
Germany	6 (6)
The Netherlands	19 (20)
Spain	5 (5)
UK	12 (13)
USA	8 (8)
Other^b^	14 (10)
Background, number (%)^c^	
Allied health care professional	30 (32)
Clinimetrician/psychometrician	29 (31)
Epidemiologist	40 (42)
Physician	28 (30)
Statistician	10 (11)
Other^d^	15 (16)
Current profession, number (%)^c^	
Clinician	26 (27)
Journal editor	9 (10)
Researcher	88 (93)
Other^e^	10 (11)
Level of experience in COS development, number (%)	
A lot	11 (12)
Some	28 (30)
A little	26 (27)
None	30 (32)
Level of experience in instrument development, number (%)	
A lot	32 (34)
Some	39 (41)
A little	12 (13)
None	12 (13)
Level of experience with evaluation of measurement properties, number (%)	
A lot	44 (46)
Some	33 (35)
A little	14 (15)
None	4 (4)
Level of experience in conducting systematic reviews, number (%)	
A lot	20 (21)
Some	35 (37)
A little	19 (20)
None	21 (22)

^a^In some cases, the total numbers are not exactly 100 % because of rounding of percentages to no decimal places

^b^Brazil (*N* = 1), France (*N* = 2), Italy (*N* = 3), Norway (*N* = 1), Portugal (*N* = 1), Switzerland (*N* = 1)

^c^As panelists could tick more than one response option, the total score exceeded 100 %

^d^Trialist (*N* = 2), systematic reviewer (*N* = 1), social research methodologist (*N* = 2), clinical academic (*N* = 1), scientific researcher (*N* = 1), health services researcher (*N* = 1), clinical psychologist (*N* = 2), project manager (*N* = 1), public health (*N* = 1), academic course writer/teacher (*N* = 1), clinical researcher (*N* = 1), human movement scientist (*N* = 1)

^e^Academic (*N* = 2), consultant for clinical researches (*N* = 1), research funder (*N* = 1), Health Technology Assessment consultant (*N* = 2), educator (*N* = 1), project manager (*N* = 1), advisor on research methods (*N* = 1), director of collaborative centre (*N* = 1)

### Delphi rounds

In the first round, panelists were asked to rate 78 questions. Consensus was reached on 58 questions (74 %).

In the second round, panelists were asked to rerate 20 questions on which no consensus was achieved in the first round. In addition, 19 new questions were formulated based on the additional comments invited in the first round. For 2/19 new questions, a 70 % or greater consensus was not reached (67 %^1^ and 48 %^2^, respectively). For 7/19 questions, consensus was reached (range 71 to 84 %) but 15 % or more of the panelists disagreed. In reviewing the panelists’ comments on these items, it was clear that for a total of eight questions we were too restrictive in our formulations, too brief in the descriptions of the tasks, or that certain tasks might not be applicable in all circumstances.

In the third round, panelists were provided with eight new formulations, instead of questions, of the paragraph for potential inclusion in the guideline intending to address nuances applicable to specific situations. For example, in the first round it was suggested that the selection of OMIs should always be guided by a review of the face validity of an OMI. In the second round, panelists were asked if COS developers themselves should assess the face validity of an outcome measurement instrument to be included in a COS. Eighty-four percent of the panelists agreed; however, 16 % of them (strongly) disagreed. It was argued that only if no face validity assessment is reported in the literature, COS developers should do it themselves. In the third round, we proposed the following recommendation for the guideline: “It is recommended that, in case no face validity assessment is reported in the literature, COS developers assess the face validity of an OMI to be included in a COS.” On all eight formulations of the paragraph for potential inclusion in the guideline consensus was reached (range 81 to 93 %), but 15 % or more of the panelists disagreed on three of these formulations (15 %, 15 %, and 19 %, respectively). As no opposing arguments were provided against these three formulations, the Steering Committee decided to include all eight proposed formulations in the guideline.

We reached consensus on four main steps in the selection of OMIs for outcomes included in a COS (Table 2). Each of these four steps includes a variety of tasks.

**Table 2:** Consensus on four main steps in the selection of outcome measurement instruments for Core Outcome Sets (COSs), including their tasks

		Percentage of agreement in the Delphi study (%)
**Step 1. Conceptual considerations**		
Aspects to consider before starting to search for outcome measurement instruments:		
1.	The construct (i.e., outcome or domain) to be measured	98
2.	The target population (e.g., age, gender, disease characteristics)	99
**Step 2. Finding existing outcome measurement instruments**		
COS developers should aim for finding *all* existing outcome measurement instruments.		72
When finding outcome measurement instruments, COS developers can have three sources of information: (1) systematic reviews, (2) literature searches, and (3) other sources (optional)		
1.	COS developers use existing, good quality, and up-to-date systematic reviews of outcome measurement instruments	94
2	a. MEDLINE (e.g., through the PubMed or OVID interface) is considered the minimum database to consult in finding all existing outcome measurement instruments. An additional search in EMBASE is highly recommended	99 and 82, respectively
	b. Reference lists of the included studies should be checked to find all existing outcome measurement instruments	91
3.	Additional sources may be considered as optional sources in finding relevant outcome measurement instruments	89
**Step 3. Quality assessment of outcome measurement instruments**		
To evaluate the quality of the outcome measurement instruments, COS developers evaluate (1) the measurement properties and (2) the feasibility aspects of the identified outcome measurement instruments		
1.	Evidence on the measurement properties should be available in the target population^a^	70–93
2.	Feasibility aspects should be taken into consideration in the selection of outcome measurement instruments for outcomes included in a COS^b^	77–97
**Step 4. Generic recommendations on the selection of outcome measurement instruments for a COS**		
1.	Select only one outcome measurement instrument for each outcome (e.g., construct or domain) in a COS	90
2.	The minimum requirements for including an outcome measurement instrument in a COS are: at least high quality evidence^c^ for good^d^ content validity and for good^d^ internal consistency (if applicable), and if the outcome measurement instrument is feasible	81
3.	A consensus procedure to agree on the outcome measurement instruments for each outcome included in a COS should be performed among all relevant stakeholders, including patients	90

^a^See Table 3 for the percentage of agreement per measurement property separately

^b^See Table 6 for the percentage of agreement per feasibility aspect separately

^c^“High quality evidence” is defined as consistent findings in multiple studies of at least good quality OR in one study of excellent quality AND a total sample size of 100 patients or more (Table 5)

^d^“Good” is defined as a “+”  rating according to the criteria for good measurement properties (Table 4)

### Step 1. Conceptual considerations

We reached 98–99 % consensus that the first step in the selection of OMIs is to agree in detail upon the construct (i.e., outcome or domain) to be measured [11] and the target population (e.g., age, gender, disease characteristics) (Table 2). This is a key task of the group developing a COS for which OMIs are sought.

### Step 2. Finding existing outcome measurement instruments

We reached 70–99 % consensus that the second step is to find existing OMIs. With the intention to search for all existing OMIs, three sources of information can be used: (1) systematic reviews, (2) literature searches, and (3) other sources, considered as optional (Table 2). The COSMIN guideline for systematic reviews of OMIs recommends that those searching the literature for all OMIs do not use search terms to cover “type of OMI” because a wide variety of terminology is used (e.g., OMIs are also termed measures, methods, questionnaires, tests, etc.). This variety of terms that has been used in the original articles can lead to a high risk of missing relevant studies [4]. There is, however, one exception for patient-reported outcome measures (PROMs): for these a comprehensive PROM filter, developed for PubMed by the Patient Reported Outcomes Measurement Group of the University of Oxford, can be used. This search filter is available through the COSMIN website [18]. In all other cases it is recommended to only use search terms for “construct,” “population,” and “measurement properties” in the search for all OMIs [4].

### Step 3. Quality assessment of outcome measurement instruments

We reached 70–97 % consensus that the third step in the selection of OMIs is quality assessment of the available OMIs. According to COSMIN, this includes two distinctive parts: (1) evaluation of the methodological quality of the included studies by using the COSMIN checklist [6] and (2) evaluation of the quality of the OMIs (i.e., their measurement properties and feasibility aspects) by applying criteria for good measurement properties (Table 2) [19].

Following the COSMIN taxonomy on which international consensus was reached [6, 13], all nine measurement properties were considered relevant in the selection process of OMIs for outcomes included in a COS (Table 3). Consensus was achieved on the criteria for good measurement properties (Table 4). The quality assessment applies to all different types of OMIs, such as assessments by health professionals, biomarkers, clinical rating scales, imaging tests, laboratory tests, patient questionnaires, and performance-based tests, and the applicable measurement properties should be evaluated.

**Table 3:** Overview of all measurement properties, including their definitions

Measurement property	Definition according to the COSMIN^a^ taxonomy	Percentage of agreement in the Delphi study (%)
Content validity (including face validity)	The degree to which the content of a measurement instrument is an adequate reflection of the construct to be measured	93
Reliability	The degree to which the measurement is free from measurement error	91
Responsiveness	The ability of a measurement instrument to detect change over time in the construct to be measured	91
Internal consistency	The degree of interrelatedness among the items	90
Structural validity	The degree to which the scores of a measurement instrument are an adequate reflection of the dimensionality of the construct to be measured	83
Measurement error	The systematic and random error of a patient’s score that is not attributed to true changes in the construct to be measured	83
Hypotheses testing	The degree to which the scores of a measurement instrument are consistent with hypotheses based on the assumption that the measurement instrument validly measures the construct to be measured	82
Criterion validity	The degree to which the scores of a measurement instrument are an adequate reflection of a “gold standard”	76
Cross-cultural validity	The degree to which the performance of the items on a translated or culturally adapted measurement instrument is an adequate reflection of the performance of the items of the original version of the measurement instrument	70

^a^ COnsensus-based Standards for the selection of health Measurement INstruments

**Table 4:** Criteria for good measurement properties

Measurement property	Rating^*^	Criteria	Percentage of agreement in the Delphi study (%)
Content validity (including face validity)	**+**	All items refer to relevant aspects of the construct to be measured AND are relevant for the target population AND are relevant for the context of use AND together comprehensively reflect the construct to be measured	97
	**?**	Not all information for ‘+’ reported	
	**–**	Criteria for ‘+’ not met	
Structural validity	**+**	**CTT:** Unidimensionality: EFA: First factor accounts for at least 20% of the variability AND ratio of the variance explained by the first to the second factor greater than 4 OR Bi-factor model: Standardized loadings on a common factor >0.30 AND correlation between individual scores under a bi-factor and unidimensional model >0.90 Structural validity: CFI or TLI or comparable measure >0.95 AND RMSEA <0.06 OR SRMR <0.08	CTT: 84 Rasch/IRT: 90
		**Rasch/IRT:** At least limited evidence for unidimensionality or positive structural validity AND no evidence for violation of local independence: Rasch: standardized item-person fit residuals between -2.5 and 2.5; OR IRT: residual correlations among the items after controlling for the dominant factor < 0.20 OR Q3's < 0.37 AND no evidence for violation of monotonicity: adequate looking graphs OR item scalability >0.30 AND adequate model fit: Rasch: infit and outfit mean squares ≥ 0.5 and ≤ 1.5 OR Z-standardized values > -2 and <2; OR IRT: G^2^ >0.01; Optional additional evidence: Adequate targeting; Rasch: adequate person-item threshold distribution; IRT: adequate threshold range No important DIF for relevant subject characteristics (such as age, gender, education), McFadden's R^2^ < 0.02	
	**?**	CTT: Not all information for ‘+’ reported IRT: Model fit not reported	
	**–**	Criteria for ‘+’ not met	
Internal consistency	**+**	At least limited evidence for unidimensionality or positive structural validity AND Cronbach's alpha(s) ≥ 0.70 and ≤ 0.95	89
	**?**	Not all information for ‘+’ reported OR conflicting evidence for unidimensionality or structural validity OR evidence for lack of unidimensionality or negative structural validity	
	**–**	Criteria for ‘+’ not met	
Reliability	**+**	ICC or weighted Kappa ≥ 0.70	88
	**?**	ICC or weighted Kappa not reported	
	**–**	Criteria for ‘+’ not met	
Measurement error	**+**	SDC or LoA < MIC	72
	**?**	MIC not defined	
	**–**	Criteria for ‘+’ not met	
Hypotheses testing	**+**	At least 75% of the results are in accordance with the hypotheses	87
	**?**	No correlations with instrument(s) measuring related construct(s) AND no differences between relevant groups reported	
	**–**	Criteria for ‘+’ not met	
	**+**	No important differences found between language versions in multiple group factor analysis or DIF analysis	
Cross-cultural validity	**?**	Multiple group factor analysis AND DIF analysis not performed	84
	**–**	One or more criteria for ‘+’ not met	
Criterion validity	**+**	Convincing arguments that gold standard is “gold” AND correlation with gold standard ≥ 0.70	88
	**?**	Not all information for ‘+’ reported	
	**–**	Criteria for ‘+’ not met	
Responsiveness	**+**	At least 75% of the results are in accordance with the hypotheses	88
	**?**	No correlations with changes in instrument(s) measuring related construct(s) AND no differences between changes in relevant groups reported	
	**–**	Criteria for ‘+’ not met	

Modified from Terwee et al. [19]

AUC = area under the curve, CFI = comparative fit index, CTT = classical test theory, DIF = differential item functioning, EFA = exploratory factor analysis, ICC = intraclass correlation coefficient, IRT = item response theory, LoA = limits of agreement, MIC = minimal important change, RMSEA = root mean square error of approximation, SEM = Standard Error of Measurement, SDC = smallest detectable change, SRMR = standardized root mean residuals, TLI = Tucker-Lewis index

^*^ “+” = positive rating, “?” = indeterminate rating,” –“ = negative rating

In the evaluation of the measurement properties of the OMIs that could potentially be included in a COS, COSMIN recommends a predefined order of importance of evaluating the measurement properties: (1) content validity, (2) internal structure (i.e., structural validity and internal consistency, and/or Item Response Theory (IRT)/Rasch model fit), and where applicable (3) the remaining measurement properties (i.e., reliability, measurement error, hypotheses testing, cross-cultural validity, criterion validity, and responsiveness). Content validity is considered to be the most important measurement property of an OMI because if it is unclear what the OMI is actually measuring, the assessment of the other measurement properties is not valuable. If the content validity of an OMI is poor or unknown, the OMI will not be further considered in the selection process. Subsequently, the internal structure (i.e., internal consistency and structural validity) should be evaluated. In case there is evidence that the internal structure of an OMI is poor, the OMI will not be further considered, i.e., the other measurement properties (including reliability, measurement error, hypotheses testing, cross-cultural validity, criterion validity, and responsiveness) will not be further evaluated [4].

To reach a conclusion about the overall quality of an OMI, an overall evaluation of the OMI should be constructed, based on all available evidence [20]. This can be done by a best-evidence synthesis, where the quality of evidence should be graded for a body of evidence for each measurement property, taking into account the number of studies, the methodological quality of the studies, and the consistency of the results of the measurement properties (Table 5) [4].

**Table 5:** Quality of evidence

Quality rating	Criteria
High	Consistent findings in multiple studies of at least good quality OR one study of excellent quality AND a total sample size of ≥100 patients
Moderate	Conflicting findings in multiple studies of at least good quality OR consistent findings in multiple studies of at least fair quality OR one study of good quality AND a total sample size of ≥50 patients
Low	Conflicting findings in multiple studies of at least fair quality OR one study of fair quality AND a total sample size of ≥30 patients
Very low	Only studies of poor quality OR a total sample size of <30 patients
Unknown	No studies

We reached 77–97 % consensus that COS developers should take feasibility aspects into consideration in the selection of OMIs for outcomes included in a COS (Table 6).

**Table 6:** Overview of all feasibility aspects

Feasibility aspects	Percentage of agreement in the Delphi study (%)
Patient’s comprehensibility	97
Interpretability	95
Ease of administration	93
Length of the outcome measurement instrument	91
Completion time	91
Patient’s mental ability level	91
Ease of standardization	90
Clinician’s comprehensibility	90
Type of outcome measurement instrument	90
Cost of an outcome measurement instrument	89
Required equipment	88
Type of administration	87
Availability in different settings	86
Copyright	85
Patient’s physical ability level	85
Regulatory agency’s requirement for approval	84
Ease of score calculation	77

### Step 4. Generic recommendations on the selection of outcome measurement instruments for a COS

We reached 81–90 % consensus on three generic recommendations concerning the final decision-making on including an OMI in a COS: (1) it is recommended to select only one OMI for each outcome (i.e., constructs or domains) in a COS, which will enhance the comparability of clinical trials, (2) it is recommended that an OMI can be provisionally included in a COS if there is at least high quality evidence^3^ for good^4^ content validity and good^4^ internal consistency (or evidence for test-retest or interrater reliability) and if the OMI is feasible, and (3) it is recommended that COS developers use a consensus procedure to get final agreement on the selected OMIs included in a COS among relevant stakeholders, including patients (Table 2).

Following the *OMERACT Handbook*, the next phase of research needs to be more explicit on what categories of stakeholders should be considered (patients, public, practitioner, press, policy-maker, program manager, professor, payer) and what the minimum requirements are for consensus [10, 11].

In addition, we reached 95 % consensus that, in general, the methods for the selection of OMIs for a COS are considered to be similar to the methods for selecting OMIs for individual clinical trials. However, as in practice it may not be feasible to perform all these steps for a clinical trial, trialists can then chose to use those OMIs that are included in a COS.

The four main steps, including their tasks, were included in the final guideline that can be found in Additional file 2.

## Discussion

The present guideline on methods for selecting OMIs can be used by COS developers in defining *how* to measure the core outcomes (i.e., constructs or domains) that are included in a COS. The guideline is based on the results of the Delphi study, the methodology derived from the COSMIN initiative, and recommendations from OMERACT [11]. With this stepwise approach, we intend to optimize the methodology of selecting OMIs for outcomes included in a COS. The field of COS development is relatively new but rapidly growing; COMET maintains a database with the aim of including all registered and ongoing initiatives on COS development including, for example, the Harmonizing Outcome Measures for Eczema (HOME), and the Initiative on Methods, Measurement, and Pain Assessment in Clinical Trials (IMMPACT) initiatives. Currently, this database includes 249 published COS studies that relate to 300 COS, and 144 ongoing COS studies [5, 21]. Other examples of the potential impact of COSs are that the National Institute for Health Research’s (a UK research funding body) Health Technology Assessment program, requires COSs to be considered in the funding applications of clinical trials, and that Cochrane and Grading of Recommendations Assessment, Development and Evaluation (GRADE) are encouraging the use of COSs in reviews and clinical practice guidelines. We believe that methodology guidelines should be based on the agreed methodology so as to deliver high-quality COSs that can be used in future clinical trials and other research. Using high-quality COSs will ultimately improve the conduct and reporting of clinical trials, enhance the value of evidence synthesis by reducing heterogeneity between trials, and may reduce outcome reporting bias. COSs reflect the best evidence at the time. However, as the field of COS development is continuously evolving (e.g., existing OMIs are further tested and new ones are being developed), the OMIs included in a COS might be reconsidered and/or replaced in light of new evidence.

There may be good reasons for COS developers to deviate from the guideline. For example, OMERACT wants responsiveness to be assessed before inclusion in a provisional core set, whereas we reached consensus for at least high quality evidence for good content validity and for good internal consistency. Another example is that, although a Cronbach’s alpha of >0.95 usually indicates item redundancy, there may be good reasons to retain certain potentially redundant items in a questionnaire. Also, we realize that in practice not all steps might be feasible within a given time frame or budget. We recommend that COS developers should decide what is feasible in their time frame and within their budget.

Although the methods for the selection of OMIs for a COS are considered to be similar to the methods for selecting OMIs for individual clinical trials, it was argued that a higher standard for selecting OMIs for a COS may be justified. Furthermore, it may not be feasible to perform all these steps for a clinical trial. This underlines the importance of the development of COSs, as trialists can then chose to use those OMIs that are included in a COS. When the primary outcome of a clinical trial is not a core outcome, the COS still needs to be measured. However, trialists could apply these recommendations to select the OMI for their primary outcome.

We acknowledge the limitations that might arise because of the relatively low response rate to the initial invitation of our Delphi study. As the results of Delphi studies in general are highly dependent upon the composition of the panel, we aimed to include a sample of experts who represent diverse disciplines, institutes and organizations and reflect the population that is intended to use a guideline for OMI selection. However, it is difficult to examine the representativeness of the panelists as it is impossible to draw a random sample from all experts. Experts were, therefore, selected nonsystematically, which may be considered as a limitation of our Delphi study. Another limitation of our study is that we did not include patient research partners in the Delphi process. We acknowledge that, herewith, we may have omitted their contribution to the selection of OMIs.

## Conclusions

This consensus-based guideline on the methods for selecting OMIs for outcomes included in a COS can be used by COS developers and clinical trialists to define *how* to measure core outcomes (i.e., constructs or domains) for any diseases or other condition in health and social care.

## Additional files

Additional file 1: Search strategies for MEDLINE, EMBASE, PsycINFO and Cinahl. (DOCX 19 kb) Additional file 2: Guideline for selecting outcome measurement instruments for outcomes included in a COS. (PDF 194 kb)

## Abbreviations

COMET: Core Outcome Measures in Effectiveness Trials Initiative
COS: Core Outcome Set
COSMIN: COnsensus-based Standards for the selection of health Measurement INstruments
OMERACT: Outcome Measures in Rheumatology
OMI: Outcome Measurement Instrument

## Footnotes

1 Question: “The minimum standard for internal consistency of outcome measurement instruments to be included in a COS should be a Cronbach’s alpha of between 0.70 and 0.90.”

2 Question: “If no outcome measurement instrument exists that meets the requirements for adequate measurement properties, it can be included in a COS “conditionally.” What should be the minimum condition before an instrument can be included in a COS?”

3 “High quality evidence” is defined as consistent findings in multiple studies of at least good quality OR in one study of excellent quality AND a total sample size of 100 patients or more (Table 5)

4 “Good” is defined as a “+” rating according to the criteria for good measurement properties (Table 4)
