# Supplementary material for: How to select outcome measurement instruments for outcomes included in a “Core Outcome Set” – a practical guideline
Source: Trials. 2016 Sep 13;17(1):449. doi: 10.1186/s13063-016-1555-2 (PMC5020549; doi:10.1186/s13063-016-1555-2)
Supplement: Additional file 1: — Search strategies for MEDLINE, EMBASE, PsycINFO and Cinahl. (DOCX 19 kb) [file 13063_2016_1555_MOESM1_ESM.docx]

**Additional file 1. Search strategies for MEDLINE, EMBASE, PsycINFO, and Cinahl**

|  | Search terms | | |
| --- | --- | --- | --- |
|  | MEDLINE | | |
| 1. | “Outcome Assessment (Health Care)”/ | | |
| 2. | Clinical Trials as Topic/ | | |
| 3. | guidance.ti,kw. | | |
| 4. | guideline*.ti,kw. | | |
| 5. | (outcome* adj2 measure*).ti,kw. | | |
| 6. | outcome*.ti,kw. | | |
| 7. | treatment outcome.ti,kw. | | |
| 8. | outcome studies.ti,kw. | | |
| 9. | outcomes assessment.ti,kw. | | |
| 10. | (outcome* adj2 instrument*).ti,kw. | | |
| 11. | measurement instrument*.ti,kw. | | |
| 12. | 1 or 2 | | |
| 13. | 3 or 4 or 5 or 6 or 7 or 8 or 9 or 10 or 11 | | |
| 14. | 12 and 13 | | |
| 15. | limit 14 to (guideline or meta analysis or “review”) | | |
| 16. | limit 15 to English language | | |
| 17. | limit 16 to humans | | |
|  | EMBASE | | |
| 1. | outcome assessment/ | | |
| 2. | (outcome* adj2 measure*).ti,kw. | | |
| 3. | outcome studies.ti,kw. | | |
| 4. | outcome* assessment.ti,kw. | | |
| 5. | (outcome* adj2 instrument*).ti,kw. | | |
| 6. | measurement instrument*.ti,kw. | | |
| 7. | 2 or 3 or 4 or 5 or 6 | | |
| 8. | 1 and 7 | | |
| 9. | limit 8 to (meta analysis or outcomes research or "systematic review") | | |
| 10. | limit 9 to (human and english language) | | |
|  | PsycINFO | | |
| 1. | treatment outcomes/ | | |
| 2. | outcome [assessment.mp](http://assessment.mp). | | |
| 3. | outcome [studies.mp](http://studies.mp). | | |
| 4. | (outcome* adj2 instrument*).mp. [mp=title, abstract, heading word, table of contents, key concepts, original title, tests & measures] | | |
| 5. | measurement instrument*.mp. | | |
| 6. | 2 or 3 or 4 or 5 | | |
| 7. | 1 and 6 | | |
| 8. | guidance.ab. | | |
| 9. | guideline*.ab. | | |
| 10. | standard*.ab. | | |
| 11. | 8 or 9 or 10 | | |
| 12. | 7 and 11 | | |
| 13. | limit 12 to (human and english language) | | |
|  | Cinahl | | |
| S1 | MM treatment outcomes | Search modes - Find all my search terms | Interface - EBSCOhost  Search Screen - Advanced Search  Database - CINAHL Plus with Full Text |
| S2 | MM outcome assessment | Search modes - Find all my search terms | Interface - EBSCOhost  Search Screen - Advanced Search  Database - CINAHL Plus with Full Text |
| S3 | AB outcome studies | Search modes - Find all my search terms | Interface - EBSCOhost  Search Screen - Advanced Search  Database - CINAHL Plus with Full Text |
| S4 | AB outcome* instrument* | Search modes - Find all my search terms | Interface - EBSCOhost  Search Screen - Advanced Search  Database - CINAHL Plus with Full Text |
| S5 | AB measurement instrument* | Search modes - Find all my search terms | Interface - EBSCOhost  Search Screen - Advanced Search  Database - CINAHL Plus with Full Text |
| S6 | AB Guidance | Search modes - Find all my search terms | Interface - EBSCOhost  Search Screen - Advanced Search  Database - CINAHL Plus with Full Text |
| S7 | MM Systematic review | Search modes - Find all my search terms | Interface - EBSCOhost  Search Screen - Advanced Search  Database - CINAHL Plus with Full Text |
| S8 | AB Meta-analysis | Search modes - Find all my search terms | Interface - EBSCOhost  Search Screen - Advanced Search  Database - CINAHL Plus with Full Text |
| S9 | S1 OR S2 OR S3 OR S4 OR S5 | Search modes - Find all my search terms | Interface - EBSCOhost  Search Screen - Advanced Search  Database - CINAHL Plus with Full Text |
| S10 | S8 OR S7 OR S6 | Search modes - Find all my search terms | Interface - EBSCOhost  Search Screen - Advanced Search  Database - CINAHL Plus with Full Text |
| S11 | S9 AND S10 | Search modes - Find all my search terms | Interface - EBSCOhost  Search Screen - Advanced Search  Database - CINAHL Plus with Full Text |
| S12 | S9 AND S10 | Limiters - English Language  Search modes - Find all my search terms | Interface - EBSCOhost  Search Screen - Advanced Search  Database - CINAHL Plus with Full Text |
| S13 | S9 AND S10 | Limiters - Scholarly (Peer Reviewed) Journals; English Language  Search modes - Find all my search terms | Interface - EBSCOhost  Search Screen - Advanced Search  Database - CINAHL Plus with Full Text |
| S14 | S9 AND S10 | Limiters - Scholarly (Peer Reviewed) Journals; English Language  Narrow by SubjectMajor: - treatment outcomes  Search modes - Find all my search terms | Interface - EBSCOhost  Search Screen - Advanced Search  Database - CINAHL Plus with Full Text |
