# Supplementary material for: How to select outcome measurement instruments for outcomes included in a “Core Outcome Set” – a practical guideline
Source: Trials. 2016 Sep 13;17(1):449. doi: 10.1186/s13063-016-1555-2 (PMC5020549; doi:10.1186/s13063-016-1555-2)
Supplement: Additional file 2: — Guideline for selecting outcome measurement instruments for outcomes included in a COS. (PDF 194 kb) [file 13063_2016_1555_MOESM2_ESM.pdf]

Guideline for selecting outcome measurement instruments for outcomes included in a Core Outcome Set

Cecilia AC Prinsen[1], Sunita Vohra[2], Michael R Rose[3], Maarten Boers[1,4], Peter Tugwell[5], Mike Clarke[6], Paula R Williamson[7], Caroline B Terwee[1]

[1]VU University Medical Center, Department of Epidemiology and Biostatistics, EMGO Institute for Health and Care Research, The Netherlands (c.prinsen@vumc.nl; cb.terwee@vumc.nl; m.boers@vumc.nl)

[2]Department of Pediatrics, Faculty of Medicine and Dentistry, and School of Public Health, and Women's and Children's Health Research Institute, University of Alberta, Canada (svohra@ualberta.ca)

[3]Department of Neurology, King's College Hospital, London, United Kingdom (m.r.rose@kcl.ac.uk)

[4] Amsterdam Rheumatology & Immunology Center, Amsterdam, The Netherlands (m.boers@vumc.nl)

[5] Department of Medicine, University of Ottawa, Ottawa, Canada (Tugwell.BB@uOttawa.ca)

[6] Queens University Belfast, All-Ireland Hub for Trials Methodology Research, Institute of Clinical Sciences, Royal Victoria Hospital, Belfast, UK (m.clarke@qub.ac.uk)

[7] Department of Biostatistics, University of Liverpool, Liverpool, UK (P.R.Williamson@liverpool.ac.uk)

Final version (dated 05-Sep-2016)

Table of Contents

Abbreviations and definitions 3

Introduction 4

Step 1. Conceptual considerations 5

Step 2. Finding existing outcome measurement instruments 5

Systematic reviews 5

Literature searches 6

Other sources 7

Step 3. Quality assessment of outcome measurement instruments 8

Evaluation of the methodological quality of the included studies 8

Evaluation of the quality of the measurement properties 9

Best evidence synthesis 14

Feasibility aspects 15

Step 4. Generic recommendations on the selection of outcome measurement instruments for a COS 16

Select only one outcome measurement instrument for each outcome in a COS 16

Minimum requirements for including an outcome measurement instrument in a COS 17

A consensus procedure to agree on the outcome measurement instrument for each outcome in a COS 17

Summary 18

Funding 20

Acknowledgements 20

References 21

Abbreviations and definitions

COMET Initiative

Core Outcome Measures in Effectiveness Trials Initiative

Core Outcome Set (COS)

A Core Outcome Set (COS) is an agreed standardized set of outcomes that should be measured and reported, as a minimum, in all clinical trials in a specific disease or trial population. A COS may be disease or population specific, covering a subset of or all interventions, but is not trial specific. This means that core outcomes should be measured in all trials conducted in that disease/population, but they do not necessarily have to include the primary outcome of the trial as this is trial specific.

COSMIN

COnsensus-based Standards for the selection of health Measurement INstruments

OMERACT

Outcome Measures in Rheumatology

Outcome

An outcome refers to *what* is being measured. It is also referred to as a construct or domain. In the context of a clinical trial it refers to what is being measured on trial participants to examine the effect of exposure to a health intervention.

Outcome Measurement Instrument (OMI)

An outcome measurement instrument refers to *how* the outcome is being measured. It is a tool to measure a quality or quantity of the outcome. The tool can be a single question, a questionnaire, a score obtained through physical examination, a laboratory measurement, a score obtained through observation of an image, etcetera.

Introduction

A joint initiative between the COnsensus-based Standards for the selection of health Measurement INstruments (COSMIN) initiative[1] and the Core Outcome Measures in Effectiveness Trials (COMET) initiative[2] aimed to develop a guideline on how to select outcome measurement instruments (OMIs) (e.g. assessments by health professionals, biomarkers, clinical rating scales, imaging tests, laboratory tests, patient questionnaires, and performance-based tests) for outcomes (i.e. constructs or domains) included in a Core Outcome Set (COS). A COS is an agreed minimum set of outcomes that should be measured and reported in all clinical trials of a specific disease or trial population; it is a recommendation of *what* should be measured and reported in all clinical trials.[3] Once a COS is defined, it is then important to achieve consensus on *how* these outcomes should be measured, i.e. which OMIs should be selected.

The present guideline results from an International Delphi study, among different groups of stakeholders, that took place between November 2013 and October 2014. With this Delphi study we (that is: COSMIN and COMET) reached consensus on the different steps to be taken in the selection of OMIs for outcomes included in a COS. Details on the methods have been published elsewhere [4] and results of the Delphi study have been published separately.[*Trials* 2016, in press]

In this guideline we intended to describe the optimal methodology, i.e. the preferred approach for selecting OMIs for outcomes included in a COS. We assume the situation where choices regarding *what* to measure (i.e. the core outcomes in a COS) already have been made.

The guideline consists of four main steps to be taken in the selection of OMIs for outcomes included in a COS. Guidance on *how* OMIs should be selected is supported by other relevant sources as well, such as the methodology that has been developed by COSMIN for performing a systematic review of OMIs;[1] the Outcome Measures in Rheumatology (OMERACT) Filter 2.0 and the OMEACRT handbook for developing COS for rheumatic diseases;[5,6] and the Primary Outcomes Reporting in Trials (PORTal) initiative which looks at primary outcomes reported in adult and pediatric clinical trials.[7] We believe that a consensus-based guideline on OMI selection will enhance the development and use of COS with the advantage of improving the standards of all clinical trials.

Step 1. Conceptual considerations

The first step in the selection of OMIs for outcomes included in a COS is to agree in detail upon the construct (i.e. outcome or domain) to be measured and the target population (e.g. age, gender, disease characteristics) before starting to search for OMIs. A detailed definition of the construct and the target population, that are based on the context of use of a COS (i.e., the specific area of health or healthcare to which a COS is to apply), is a prerequisite for selecting an appropriate OMI.[8] For example, a core outcome could be 'pain'. In order to select an appropriate OMI to measure pain, a more detailed description of the construct 'pain' is warranted. For example, whether the interest is in pain intensity, pain interfering with activity, etcetera.

With regard to the target population, we recommend consideration of any relevant subgroups by age, gender, or disease characteristics that may require separate OMIs. For example, adults versus children, acute versus chronic disease, etcetera. In addition, we recommend consideration of whether separate OMIs may be required for a different context of use. For example, inpatient or outpatient setting, different administration modes, etcetera.

In general, we advise against choosing a specific type of OMI, such as assessments by health professionals, biomarkers, clinical rating scales, imaging tests, laboratory tests, patient questionnaires, and performance-based tests, before starting to search for OMIs (but with possible specific exceptions as mentioned below). It is recommended to keep the search as broad and inclusive as possible at this stage to ensure that all available OMIs are identified for further evaluation.

Step 2. Finding existing outcome measurement instruments

The aim of the second step in the selection of OMIs for outcomes included in a COS is finding existing OMIs. With the intention to search for *all* existing OMIs, three sources of information can be used: 1) systematic reviews, 2) literature searches, and 3) other sources, considered as optional.

Systematic reviews

We recommend that COS developers use existing, good quality, and up-to-date systematic reviews of OMIs for the selection of OMIs for a COS. To see if a systematic review exists, the COSMIN database of systematic reviews of OMIs can be consulted

(http://database.cosmin.nl/). This database currently contains 572 unique systematic reviews (last update: June 2015) and is updated annually.

A good quality systematic review comprises of a comprehensive, systematic literature search and should be up-to-date; the methodological quality of the included studies should have been evaluated; and the quality of all relevant OMIs should have been evaluated. COS developers should verify whether these sources of information are available or whether they need to perform or update these tasks. For example, if the literature search is up-to-date but the quality of the included studies is not assessed, we recommend that COS developers perform this task. If a systematic review exists but it is not up-to-date, we recommend that COS developers update the literature search (see below), and evaluate the methodological quality of the studies and the quality of the OMIs (Step 3). The decision for a search update will depend on how active a research area has been over the last year(s) and whether new OMIs have been developed recently. If no systematic review exists at all, we recommend that COS developers perform a systematic literature search (see below) and continue with Step 3 'Quality assessment of outcome measurement instruments'.

COS developers may decide to publish the systematic literature search as a systematic review (optional). In that case, we refer to the COSMIN protocol on how to perform a systematic review of OMIs that can be found on the COSMIN website (www.cosmin.nl).[1] The COSMIN group is currently updating this protocol, aiming to publish it as a peer-reviewed guideline for systematic reviews of OMIs.[manuscript in preparation]

Literature searches

To find existing OMIs, a comprehensive literature search is an important prerequisite and consists of blocks of search terms for the following four key elements: 1) the construct of interest; 2) the target population; 3) the type of OMI, but preferably only where the focus is on patient-reported outcome measures (PROMs), and 4) the measurement properties on which the review focuses (e.g., reliability, validity, responsiveness).[4] The COSMIN guideline for systematic reviews of OMIs recommends that those searching the literature for all OMIs do not use search terms to cover 'type of OMI' because of a high risk of missing relevant studies as many studies are not indexed as such.[1] In addition, a wide variety of terminology is being used (e.g. OMIs are also termed measures, methods, questionnaires, tests, tools, etcetera). This can also lead to a high risk of missing relevant studies. When no search terms for 'type of OMI' are included, it makes the search maximally inclusive. There is, however, one

exception for patient-reported outcome measures (PROMs): for these a comprehensive PROM filter, developed for PubMed by the Patient Reported Outcomes Measurement Group of the University of Oxford, can be used. This search filter is available through the COSMIN website.[9] With regard to search terms for 'measurement properties', a highly sensitive, validated search filter for finding studies on measurement properties can be used that was published by Terwee *et al.* (2009).[10]

MEDLINE (e.g., through the PubMed or OVID interface) is considered the minimum database that COS developers should consult for finding existing OMIs. An additional search in EMBASE is highly recommended because in several systematic reviews of OMIs, it appeared that two or three relevant articles were found in EMBASE that were not found in MEDLINE.[11-13] We also recommend searching in other specific databases as well, depending on the construct and target population of interest, for example the Cochrane Library, Cinahl, or PsycINFO. Lastly, we recommend that the reference lists of the included studies should be screened for additional relevant studies.[1]

Other sources

Additional sources of information that can be consulted for finding existing OMIs include (online) databases of OMIs, books and/or book chapters, conference proceedings, contact/-lead authors of publications in the field, World Wide Web, trial registries, citations, consumer networks, patient organizations, and special interest groups. Examples of relevant online databases can be found in Table 1. However, searches in these additional sources cannot be performed in a systematic and reproducible manner. Moreover, it is unlikely that one will find OMIs of good quality that were not already identified in a systematic literature search. We therefore consider such searches as optional.

Name database	URL
COSMIN database of systematic reviews of outcome measurement instruments	http://database.cosmin.nl/
Health and Psychosocial Instruments database	http://www.ebscohost.com/academic/health-and-psychosocial-instruments-hapi

IN-CAM database	http://www.incamresearch.ca/content/welcome-cam-health-outcomes-database
Measurement Instrument Database for the Social Sciences	http://www.midss.ie/
PROQOLID database	http://www.proqolid.org/
Registry of Outcome Measures	http://www.researchrom.com/

Table 1. Examples of relevant online databases

Step 3. Quality assessment of outcome measurement instruments

The third step in the selection of OMIs is a quality assessment of the OMIs that result from Step 2. There are many aspects to assessing the measurement properties of OMIs and their constructs and definitions do vary. We chose the definitions listed at http://www.cosmin.nl/COSMIN%20taxonomy.html which have been derived from an international Delphi consensus process.[14,15] The quality assessment includes two distinctive parts: 1) the evaluation of the methodological quality of the included studies by using the COSMIN checklist,[14] and 2) the evaluation of the quality of the OMIs (i.e., their measurement properties and feasibility aspects) by applying criteria for good measurement properties.[16]

Evidence on the methodological quality of the studies and the quality of the measurement properties should be combined in a best evidence synthesis, with feasibility aspects also taken into consideration. It is possible that Step 3 may already have been performed in an existing and up-to-date systematic review of good quality.

Evaluation of the methodological quality of the included studies

The COSMIN checklist is recommended to evaluate the methodological quality of the identified studies. Although the COSMIN checklist was originally developed for evaluating the quality of studies on the measurement properties of health measurement instruments, it has also been used for evaluating the quality of studies on the measurement properties of other OMIs, such as performance based tests.[17] This checklist is applied to each study and covers nine different measurement properties each rated from excellent to poor, resulting in an

overall quality score for each measurement property.[18] The COSMIN checklist, including detailed information on using the checklist, can be found on the COSMIN website.[1]

Evaluation of the quality of the measurement properties

To determine whether an OMI has good measurement properties, criteria for good measurement properties can be applied.[16] Table 2 provides a complete overview of all measurement properties, including their definitions using the COSMIN taxonomy on which international consensus was reached,[15] that are considered to be relevant in the quality assessment of OMIs. Not all measurement properties, however, apply to all kinds of OMIs. For example, internal consistency and structural validity are not relevant for laboratory tests or performance-based tests, as these measurement properties are only relevant for OMIs that are based on a reflective model (i.e., a model in which all items are a manifestation of the same underlying construct).[19]

Measurement property	Definition according to COSMIN taxonomy
**Reliability**	
Internal consistency	The degree of interrelatedness among the items
Reliability	The degree to which the measurement is free from measurement error
Measurement error	The systematic and random error of a patient's score that is not attributed to true changes in the construct to be measured
**Validity**	
Content validity (including face validity)	The degree to which the content of a measurement instrument is an adequate reflection of the construct to be measured
Structural validity	The degree to which the scores of a measurement instrument are an adequate reflection of the dimensionality of the construct to be measured
Hypotheses testing	The degree to which the scores of a measurement instrument are consistent with hypotheses based on the assumption that the measurement instrument validly measures the construct to be measured
Cross cultural validity	The degree to which the performance of the items on a translated or culturally adapted measurement instrument are an adequate reflection of the performance of the items of the original version of the measurement instrument
Criterion validity	The degree to which the scores of a measurement instrument are an adequate reflection of a 'gold standard'

**Responsiveness**	
Responsiveness	The ability of a measurement instrument to detect change over time in the construct to be measured

Table 2. Overview of all measurement properties, including their definitions

In the evaluation of the measurement properties of the OMIs potentially included in a COS, COSMIN recommends a predefined order of importance of evaluating the measurement properties: 1) content validity; 2) internal structure (i.e., structural validity and internal consistency, and/or Item Response Theory (IRT)/Rasch model fit); and where applicable 3) the remaining measurement properties (i.e. reliability, measurement error, hypotheses testing, cross-cultural validity, criterion validity, and responsiveness).[1]

Content validity (including face validity)

We recommend that the content validity (including face validity) of the included OMIs be evaluated first. Content validity is considered to be the most important measurement property of an OMI because if it is unclear what the OMI is actually measuring, the assessment of the other measurement properties is not valuable.[1] Content validity consists of a subjective judgment about the comprehensibility (i.e., the degree to which the items or tests are correctly understood by the patients), comprehensiveness (i.e., the degree to which no important items or tests are missing), and relevance of the content of an OMI for the construct and target population of interest, and considering the context of use of a COS. Judgment is needed on four sources of information: 1) the conceptual considerations (construct, target population, and context of use), 2) the quality of the development of the OMI, 3) existing face and content validity studies found in the literature, and 4) the content of the OMI. We recommend that content validity is judged by two reviewers independently and that the perspective of patients is included as well. COSMIN is currently working on updated standards and criteria for content validity that includes these aspects (a Delphi study is currently ongoing).

The methodological quality of the studies on content validity can be evaluated by completing the COSMIN box for ‘content validity’. Subsequently, criteria for good measurement properties can be applied. Table 3 provides a complete overview of the criteria for good measurement properties. These minimum criteria for content validity are in agreement with the International Society for Quality of Life Research (ISOQOL)

recommendations and the Food and Drug Administration (FDA) guidance on patient-reported outcomes.[20,21] If the content validity of an OMI is poor[2] or unknown, the OMI will not be further considered in the selection process.[1]

**Measurement property**	**Rating***	**Criteria**
Content validity (including face validity)	+	All items refer to relevant aspects of the construct to be measured AND are relevant for the target population AND are relevant for the context of use AND together comprehensively reflect the construct to be measured
	?	Not all information for '+' reported
	–	Criteria for '+' not met
Structural validity	+	**CTT:**<br><br>Unidimensionality: EFA: First factor accounts for at least 20% of the variability AND ratio of the variance explained by the first to the second factor greater than 4 OR Bi-factor model: Standardized loadings on a common factor >0.30 AND correlation between individual scores under a bi-factor and unidimensional model >0.90<br><br>Structural validity: CFI or TLI or comparable measure >0.95 AND RMSEA <0.06 OR SRMR <0.08)
		**Rasch/IRT:**<br><br>At least limited evidence for unidimensionality or positive structural validity AND no evidence for violation of local independence: Rasch: standardized item-person fit residuals between -2.5 and 2.5; OR IRT: residual correlations among the items after controlling for the dominant factor < 0.20 OR Q3's < 0.37 AND no evidence for violation of monotonicity: adequate looking graphs OR item scalability >0.30 AND adequate model fit: Rasch: infit and outfit mean squares ≥ 0.5 and ≤ 1.5 OR Z-standardized values > -2 and <2; OR IRT: G^2 >0.01;<br><br>Optional additional evidence:<br>Adequate targeting; Rasch: adequate person-item threshold distribution; IRT: adequate threshold range<br><br>No important DIF for relevant subject characteristics (such as age, gender, education), McFadden's R^2 < 0.02

	?	CTT: Not all information for '+' reported<br>IRT: Model fit not reported
	–	Criteria for '+' not met
Internal consistency	+	At least limited evidence for unidimensionality or positive structural validity AND Cronbach's alpha(s) ≥ 0.70 and ≤ 0.95
	?	Not all information for '+' reported OR conflicting evidence for unidimensionality or structural validity OR evidence for lack of unidimensionality or negative structural validity
	–	Criteria for '+' not met
Reliability	+	ICC or weighted Kappa ≥ 0.70
	?	ICC or weighted Kappa not reported
	–	Criteria for '+' not met
Measurement error	+	SDC or LoA < MIC
	?	MIC not defined
	–	Criteria for '+' not met
Hypotheses testing	+	At least 75% of the results are in accordance with the hypotheses
	?	No correlations with instrument(s) measuring related construct(s) AND no differences between relevant groups reported
	–	Criteria for '+' not met
Cross-cultural validity	+	No important differences found between language versions in multiple group factor analysis or DIF analysis
	?	Multiple group factor analysis AND DIF analysis not performed
	–	One or more criteria for '+' not met
Criterion validity	+	Convincing arguments that gold standard is "gold" AND correlation with gold standard ≥ 0.70
	?	Not all information for '+' reported
	–	Criteria for '+' not met
Responsiveness	+	At least 75% of the results are in accordance with the hypotheses
	?	No correlations with changes in instrument(s) measuring related construct(s) AND no differences between changes in relevant

		groups reported
	–	Criteria for '+' not met

Modified from Terwee *et al.* [16]
AUC = area under the curve, CFI = comparative fit index, CTT = classical test theory, DIF = differential item functioning, EFA: exploratory factor analysis, ICC = intraclass correlation coefficient, IRT = item response theory, LoA = limits of agreement, MIC = minimal important change, RMSEA = root mean square error of approximation, SEM = Standard Error of Measurement, SDC = smallest detectable change, SRMR = standardized root mean residuals, TLI = Tucker-Lewis index

* "+" = positive rating, "?" = indeterminate rating," –" = negative rating

Table 3. Criteria for good measurement properties

Internal structure

Subsequently, we recommend that the internal structure of the included OMIs should be evaluated, focusing on the structural validity (i.e., dimensionality) and internal consistency of the OMI.

Structural validity refers to the degree to which the scores of an OMI are an adequate reflection of the dimensionality of the construct of interest.[15] Structural validity can be assessed by either factor analyses, IRT analysis, or Rasch analysis.

Internal consistency refers to the degree of interrelatedness among the items.[15] Cronbach's alpha can be used to assess the internal consistency of an OMI that has been shown to be unidimensional by factor analysis.

We recommend the methodological quality of the studies on structural validity and internal consistency be evaluated by completing the applicable COSMIN boxes for 'structural validity' and for 'internal consistency'. Subsequently, criteria for good measurement properties can be applied (Table 3). The IRT criteria for structural validity were adapted from the Patient Reported Outcomes Measurement Information System (PROMIS) Standards.[22]

The assessment of structural validity and internal consistency is only relevant for OMIs based on a reflective model.[19] These items are expected to be highly correlated and interchangeable. In case the OMI is based on a formative model (i.e., when the items together form the construct and do not need to be correlated), these measurement properties are not relevant and this task can be skipped. For example, for the ACR20, a formative model that indicates how much a person's rheumatoid arthritis has improved, the assessment of inter-rater reliability may be more relevant instead.

In case there is evidence in multiple studies (i.e., at least two studies) of good quality or in one study of excellent quality that content validity (including face validity) AND structural validity or internal consistency are poor[2], the OMI will not be further considered, i.e. the other measurement properties (including reliability, measurement error, hypotheses testing, cross-cultural validity, criterion validity, and responsiveness) will not be further evaluated.[1]

Other measurement properties

As for all other measurement properties (i.e., reliability, measurement error, hypotheses testing, cross-cultural validity, criterion validity, and responsiveness), we recommend the methodological quality of the included studies be evaluated, as well as the quality of the measurement properties. The assessment of criterion validity is usually not applicable to PROMs as, in general, no gold standard exists for PROMs. There is one exception where a shortened OMI is compared to the original long version. In that case, the original long version can be considered the gold standard.[19]

Best evidence synthesis

To come to a conclusion about the overall quality of an OMI, an overall evaluation of the OMI should be constructed, based on all available evidence.[23] This can be done by a best evidence synthesis, where the quality of evidence should be graded for a body of evidence for each measurement property, taking into account the number of studies, the methodological quality of the studies, and the (consistency of the) results of the measurement properties (Table 4).[1] In general, high quality evidence is considered to be present if there are consistent findings in multiple studies (i.e., at least two studies) of good quality OR in one study of excellent quality that the measurement property meets the criteria (Table 4). For example, there is high quality evidence for internal consistency in case of consistent findings in multiple studies of good quality, or in one study of excellent quality, that the Cronbach's alpha is at least 0.70. For rating high quality evidence for internal consistency of a (sub)scale, there should also be evidence that the (sub)scale on which the Cronbach's alpha is calculated is unidimensional. Therefore, there should also be evidence of consistent findings in multiple studies of good quality or one study of excellent quality that the (sub)scales are unidimensional. This evidence may come from different studies, for example a study in which

factor analyses was performed.[24-26] For further details on how to grade the quality of evidence we refer to the COSMIN website.[1]

The principals of the best evidence synthesis are in agreement with the Grading of Recommendations Assessment, Development and Evaluation (GRADE) working group.[27] The working group has developed a systematic and transparent approach to grading the quality of evidence and strength of recommendations. This can minimize bias and aid interpretation of expert-created medical guidelines.[27]

Quality rating	Criteria
High	Consistent findings in multiple studies of at least good quality OR one study of excellent quality AND a total sample size of ≥100 patients
Moderate	Conflicting findings in multiple studies of at least good quality OR consistent findings in multiple studies of at least fair quality OR one study of good quality AND a total sample size of ≥50 patients
Low	Conflicting findings in multiple studies of at least fair quality OR one study of fair quality AND a total sample size of ≥30 patients
Very low	Only studies of poor quality OR a total sample size of <30 patients
Unknown	No studies

Table 4. Quality of evidence

Feasibility aspects

Aspects of feasibility may play an important role in the selection of an OMI for a COS. COS developers should ask themselves the following question: “Can the measure be applied easily in its intended setting, given constraints of time, money, and interpretability?”[5,6] Aspects of feasibility may be decisive in the acceptance of the OMI by researchers. A complete overview of all feasibility aspects that COS developers may take into consideration is provided in Table 5.

Feasibility aspects
Patient's comprehensibility
Interpretability
Ease of administration
Length of the outcome measurement instrument
Completion time
Patient's mental ability level
Ease of standardization
Clinician's comprehensibility
Type of outcome measurement instrument
Cost of an outcome measurement instrument
Required equipment
Type of administration
Availability in different settings
Copyright
Patient's physical ability level
Regulatory agency's requirement for approval
Ease of score calculation

Table 5. Overview of all feasibility aspects

Step 4. Generic recommendations on the selection of outcome measurement instruments for a COS

These recommendations concern the final decision making on including an OMI in a COS.

Select only one outcome measurement instrument for each outcome in a COS

Taking all evidence of the measurement properties and feasibility aspects into consideration, and the specific situation for which the OMI is intended, we recommend -in principle- to select only one OMI for each outcome in a COS. This will enhance the comparability of future clinical trials. If the outcome of interest is a complex outcome (e.g., pain) that consists

of multiple aspects that are being measured by different OMIs (e.g., pain intensity, pain interference), we recommend that these different aspects be considered as different outcomes. In addition, we recommend COS developers to consider whether different (sub)populations may need their own OMIs to measure the same outcome. For example, a different OMI may be selected to measure pain in children and in adults.

Minimum requirements for including an outcome measurement instrument in a COS

Ideally, an OMI included in a COS has high quality evidence for all measurement properties. However, in practice, there is often unknown or (very) low evidence for some measurement properties. We recommend that an OMI can be provisionally included in a COS if there is at least high quality evidence for good[1] content validity and for good[1] internal consistency (if applicable), and if the OMI seems feasible. Conversely there should be an absence of high quality evidence that one or more other measurement properties are poor[2]. If internal consistency is not relevant, evidence for test-retest or inter-rater reliability should be available. Where an OMI lacks evidence on one or more measurement properties, we recommend proposing a research agenda for further validation studies. When no OMI with good[1] content validity is available, we recommend developing a new OMI, followed by a quality assessment of the OMI.

A consensus procedure to agree on the outcome measurement instrument for each outcome in a COS

We recommend that COS developers use a consensus procedure (e.g., a consensus meeting) to get final agreement on the selected OMIs included in a COS among all relevant stakeholders, including patients. Group discussions and a plenary discussion plus voting during a face-to-face meeting among a group of stakeholders can be used to achieve consensus on the final core set of OMIs.[5,6]

[1] ‘Good’ is defined as a “+” rating according to the criteria for good measurement properties
[2] ‘Poor’ is defined as a “-” rating according to the criteria for good measurement properties

Summary

We reached consensus on four main steps in the selection of outcome measurement instruments (OMIs) for outcomes included in a Core Outcome Set (COS): Step 1) conceptual considerations; Step 2) finding existing OMIs, by means of a systematic review and/or literature searches; Step 3) quality assessment of OMIs, by means of the evaluation of the measurement properties and feasibility aspects of the OMIs; and Step 4) generic recommendations on the selection of OMIs for outcomes included in a COS. In general, the methods for the selection of OMIs for a COS are considered to be similar to the methods for selecting OMIs for individual clinical trials. A summary of this guideline is presented in a flow chart (Figure 1).

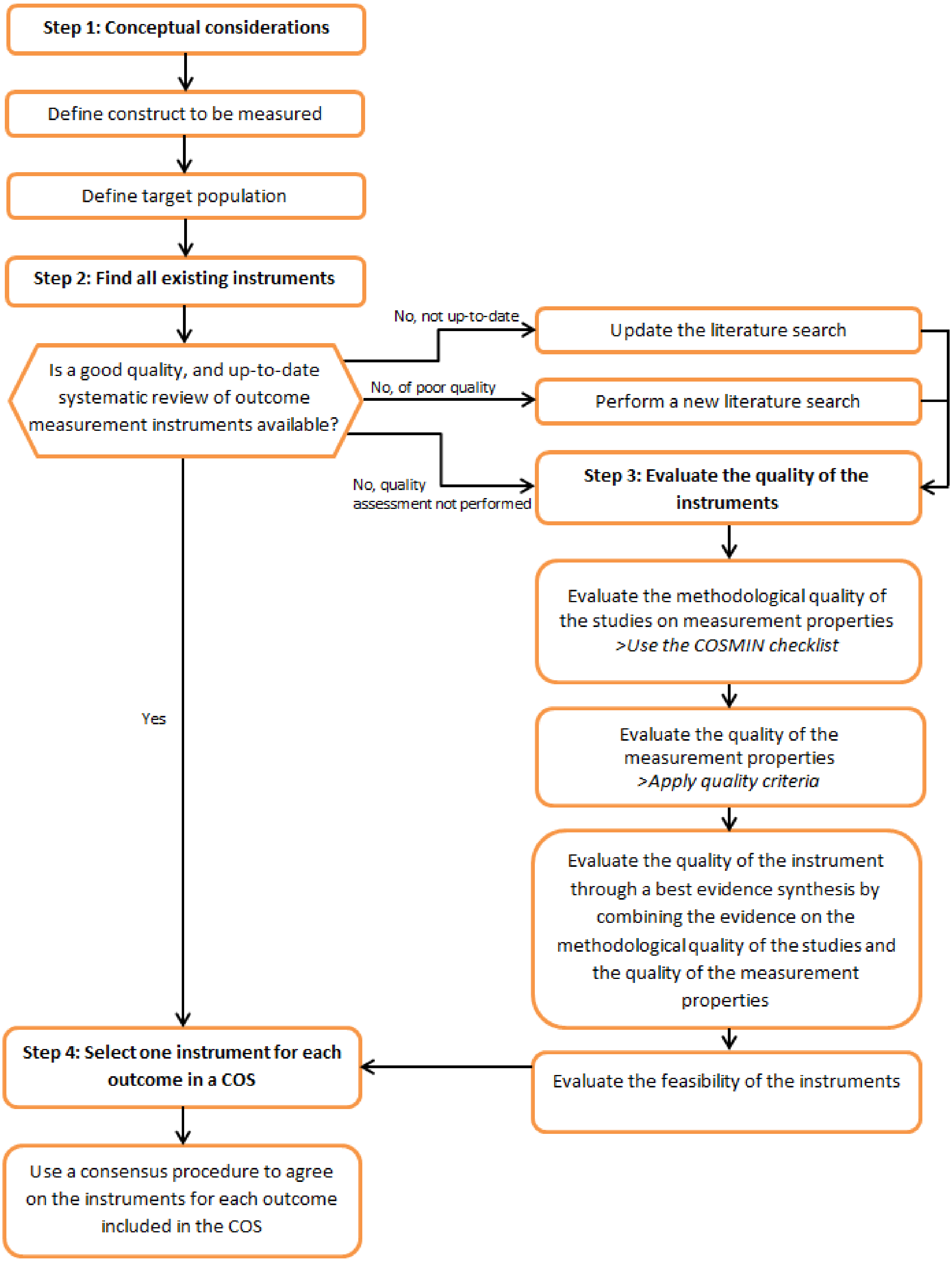

Figure 1. Flowchart for the selection of outcome measurement instruments for core outcome sets

Funding

The research has received funding from the European Union's Seventh Framework Programme (FP7/2007-2013] under grant agreement n° [305081].

Acknowledgements

We thank all panelists for their time spent completing all three Delphi questionnaires, including the following who accepted our invitation to be mentioned in the acknowledgement section:

Jordi Alonso, Felix Angst, Dorcas Beaton, Anna Beurskens, Andrea E Bialocerkowski, Jane M Blazeby, Maarten Boers, Peter J Bower, Peter Bragge, Merel Brehm, Zeeshan Butt, Patrina HY Caldwell, Sarah L Chamlin, An-Wen Chan, Alessandro Chiarotto, Kaj S Christensen, Tammy J Clifford, Natalie J Collins, Megan Davidson, Aileen M Davis, Jill Dawson, Marcel P Dijkers, Fiona L Dobson, Antonio Escobar, Ricardo M Fernandes, Montse Ferrer, Carlos G Forero, Robert Frouf, Margreth Grotle, Jan Hartvigsen, Kirstie L Haywood, Bridget E Hill, Albere JA Koke, Ida J Korfage, Jan Kottner, Johanna H van der Lee, Sarah McIntyre, Joerg J Meerpohl, Lidwine B Mokkink, Jennifer L Moore, Catherine J Morgan, Kelly K O'Brien, Amy C Plint, Marcel WM Post, Kevin Pottie, Cecilia AC Prinsen, Carlijn van Randeraad-van der Zee, Bernd Reuschenbach, Ewa M Roos, Michael R Rose, Anne WS Rutjes, Jochen Schmitt, Beverley J Shea, Lee S Simon, Peter M Smith, Claire Snyder, Sally Spencer, Mirjam AG Sprangers, David L Streiner, Nicholas F Taylor, Caroline B Terwee, Kim S Thomas, Peter Tugwell, Peter Vedsted, Henrica CW de Vet, Ester Villalonga-Olives, Sunita Vohra, Angela C Webster, Anne Whitehead, Gavin Williams, Hywel C Williams, Paula R Williamson.

References

1. COnsensus-based Standards for the selection of health Measurement INstruments. COSMIN website. [accessed 25-4-2016]. Available from http://www.cosmin.nl/

2. Core Outcome Measures in Effectiveness Trials. COMET website. [accessed 4-12-2014]. Available from http://www.comet-initiative.org/

3. Clarke M: **Standardising outcomes for clinical trials and systematic reviews.** *Trials* 2007, **8:** 39.

4. Prinsen CAC, Vohra S, Rose MR, King-Jones S, Ishaque S, Bhaloo Z *et al.*: **Core Outcome Measures in Effectiveness Trials (COMET) initiative: protocol for an international Delphi study to achieve consensus on how to select outcome measurement instruments for outcomes included in a 'core outcome set'.** *Trials* 2014, **15:** 247.

5. OMERACT Handbook. [accessed 25-4-2016]. Available from http://www.omeract.org/pdf/OMERACT_Handbook.pdf

6. Boers M, Kirwan JR, Wells GA, Beaton DE, Gossec L, D'Agostino MA *et al.*: **Developing core outcome measurement sets for clinical trials: OMERACT filter 2.0.** *J Clin Epidemiol* 2014, **67:** 745-753.

7. Adams D., Liu Y., Bhaloo Z., Hansraj N., Hartling L., Vohra S.: **Primary Outcomes Reporting in Trials (PORTal): a systematic review of pediatric randomized controlled trials.** *(accepted by J Clin Epidemiol)* 2016.

8. Williamson PR, Altman DG, Blazeby JM, Clarke M, Devane D, Gargon E *et al.*: **Developing core outcome sets for clinical trials: issues to consider.** *Trials* 2012, **13:** 132.

9. Patient Reported Outcomes Measurement Group. Nuffield Department of population Health, University of Oxford [accessed 5-9-2016]. Available from http://www.cosmin.nl/images/upload/files/PROM%20Gp%20filtersOCTOBER%202010FINAL.pdf

10. Terwee CB, Jansma EP, Riphagen II, de Vet HCW: **Development of a methodological PubMed search filter for finding studies on measurement properties of measurement instruments.** *Qual Life Res* 2009, **18:** 1115-1123.

11. de Boer MR, Moll AC, de Vet HC, Terwee CB, Volker-Dieben HJ, van Rens GH: **Psychometric properties of vision-related quality of life questionnaires: a systematic review.** *Ophthalmic Physiol Opt* 2004, **24:** 257-273.

12. Elbers RG, Rietberg MB, van Wegen EE, Verhoef J, Kramer SF, Terwee CB *et al.*: **Self-report fatigue questionnaires in multiple sclerosis, Parkinson's disease and stroke: a systematic review of measurement properties.** *Qual Life Res* 2012, **21:** 925-944.

13. Terwee CB, Bouwmeester W, van Elsland SL, de Vet HC, Dekker J: **Instruments to assess physical activity in patients with osteoarthritis of the hip or knee: a systematic review of measurement properties.** *Osteoarthritis Cartilage* 2011, **19:** 620-633.

14. Mokkink LB, Terwee CB, Patrick DL, Alonso J, Stratford PW, Knol DL *et al.*: **The COSMIN checklist for assessing the methodological quality of studies on measurement properties of health status measurement instruments: an international Delphi study.** *Qual Life Res* 2010, **19:** 539-549.

15. Mokkink LB, Terwee CB, Patrick DL, Alonso J, Stratford PW, Knol DL *et al.*: **The COSMIN study reached international consensus on taxonomy, terminology, and definitions of measurement properties for health-related patient-reported outcomes.** *J Clin Epidemiol* 2010, **63:** 737-745.

16. Terwee CB, Bot SD, de Boer MR, van der Windt DA, Knol DL, Dekker J *et al.*: **Quality criteria were proposed for measurement properties of health status questionnaires.** *J Clin Epidemiol* 2007, **60:** 34-42.

17. Dobson F, Hinman RS, Hall M, Terwee CB, Roos EM, Bennell KL: **Measurement properties of performance-based measures to assess physical function in hip and knee osteoarthritis: a systematic review.** *Osteoarthritis Cartilage* 2012, **20:** 1548-1562.

18. Terwee CB, Mokkink LB, Knol DL, Ostelo RW, Bouter LM, de Vet HCW: **Rating the methodological quality in systematic reviews of studies on measurement properties: a scoring system for the COSMIN checklist.** *Qual Life Res* 2012, **21:** 651-657.

19. COSMIN Manual. [accessed 25-4-2016] Avaiable from http://www.cosmin.nl/images/upload/files/COSMIN%20checklist%20manual%20v9.pdf

20. Reeve BB, Wyrwich KW, Wu AW, Velikova G, Terwee CB, Snyder CF *et al.*: **ISOQOL recommends minimum standards for patient-reported outcome measures used in patient-centered outcomes and comparative effectiveness research.** *Qual Life Res* 2013, **22:** 1889-1905.

21. U.S.Department of Health and Human Services Food and Drug Administration. Guidance for Industry - Patient-Reported Outcome Measures: Use in Medical Product Development to Support Labeling Claims. December 2009.

22. PROMIS Methodology. Patient Reported Outcomes Measurement Information System (PROMIS) Standards . 2015. [accessed 14-7-2015]. Available from http://www.nihpromis.org/science/methodology

23. The Standards for Educational and Psychological Testing. http://www.apa.org/science/programs/testing/standards.aspx [accessed 25-4-2016].

24. Bartels B, de Groot JF, Terwee CB: **The six-minute walk test in chronic pediatric conditions: a systematic review of measurement properties.** *Phys Ther* 2013, **93:** 529-541.

25. Conijn AP, Jens S, Terwee CB, Breek JC, Koelemay MJ: **Assessing the quality of available patient reported outcome measures for intermittent claudication: a systematic review using the COSMIN checklist.** *Eur J Vasc Endovasc Surg* 2015, **49:** 316-334.

26. Talma H, Chinapaw MJ, Bakker B, HiraSing RA, Terwee CB, Altenburg TM: **Bioelectrical impedance analysis to estimate body composition in children and adolescents: a systematic review and evidence appraisal of validity, responsiveness, reliability and measurement error.** *Obes Rev* 2013, **14:** 895-905.

27. Grading of Recommendations Assessment, Development and Evaluation. GRADE Working Group [accessed 16-4-2015]. Available from http://www.gradeworkinggroup.org
